# Prognostic and clinicopathological significance of MUC expression in head and neck cancer: a systematic review and meta-analysis

**DOI:** 10.18632/oncotarget.19648

**Published:** 2017-07-27

**Authors:** Hao Lu, Dan Liang, Yun Zhu, Wanlin Xu, Kaihua Zhou, Limin Liu, Shengwen Liu, Wenjun Yang

**Affiliations:** ^1^ Department of Oral Maxillofacial-Head and Neck Oncology, Ninth People’s Hospital, Shanghai Jiao Tong University School of Medicine, Shanghai, China; ^2^ Shanghai Research Institute of Stomatology and Shanghai Key Laboratory of Stomatology, Shanghai, China; ^3^ State Key Laboratory of Oral Diseases, National Clinical Research Center for Oral Diseases, West China Hospital of Stomatology, Sichuan University, Chengdu, China

**Keywords:** MUC, head and neck cancer, biomarkers, prognosis, meta-analysis

## Abstract

The prognostic value of mucins expression in patients with head and neck cancer (HNC) remains controversial. To address this, a meta-analysis was performed to systematically evaluate prognostic significance of mucins expression in HNC. Electronic and manual searches were performed and a total of 20 studies including 2046 patients were selected for the final analysis. Increased mucins expression was associated with unfavorable overall survival in HNC patients (HR=1.83, 95% CI: 1.43-2.33, *p*=0.000). Mucins overexpression was also in correlation with more advanced TNM stage (RR=0.84, 95% CI: 0.73-0.97, *p*=0.017), higher risk of lymph node metastasis (RR=0.69, 95% CI: 0.57-0.84, *p*=0.000) and deeper invasion (RR=0.58, 95% CI: 0.44-0.76, *p*=0.000). These results suggested that elevated mucins expression was significantly associated with worse prognosis and more detrimental clinicopathological outcomes, revealing the promising potential of mucins as biomarkers for HNC management.

## INTRODUCTION

Head and neck cancer (HNC) is a group of biologically similar cancers originating from the oral cavity, nasopharyngeal, oropharynx, hypopharynx and larynx, which mainly behave as squamous cell carcinoma histologically. HNC is the sixth most frequent type of malignant tumor, causing more than 400,000 deaths annually worldwide [1-5]. Its high mortality rate and the disfigurement or functional deficiency that survivors may suffer result in a considerable global public health burden. Despite great advance in multidisciplinary combined diagnosis and treatment, only 30-50% patients with HNC survive over 5 years after initial diagnosis worldwide [6]. Clinically, the classic tumor, node, and metastasis (TNM) staging system is widely used for the initial diagnosis but failed to reflect the inherent biological heterogeneity especially in atypical early symptoms or concealed metastasis patients. Therefore, novel biomarkers involved in cancer development are greatly needed to stratify patients with poor prognosis of HNC in order to make optimal individualized therapy.

Much attention has been focused on the involvement of mucins (MUC) in tumor carcinogenesis and metastasis recently. MUC is a family of high O-glycosylated protein, characterized by a basic structure including a central polymorphic tandem repeat region [7-8]. Only expressing on the apical surfaces of various luminal and glandular normal epithelial cells, MUC play an important role in cell-cell adhesion, immune response and alteration of intracellular signaling [9]. However, the tightly regulated homeostatic expression may be disrupted by various factors such as cancer cells, in particular. The observation of subcellular distribution and biochemical features changes during malignant transformation and tumor progression suggest that MUC may be the key point in carcinogenesis and subsequent metastasis of cancers [10-14]. Therefore, aberrant MUC expression may be predictive biomarkers in HNC.

Over the past decade, numerous independent studies have evaluated the clinical and the prognostic value of MUC protein expression in HNC. Yet, the results of these reports remain controversial and no clear consensus has been achieved so far [15-19]. Limited to small sample size some publications may draw inconsistent results due to potential random errors. Therefore, we conducted a systematic review and meta-analysis to address the association between MUC expression and prognostic value and the common clinicopathological parameters of HNC.

## RESULTS

### Eligible studies

**Figure 1:**
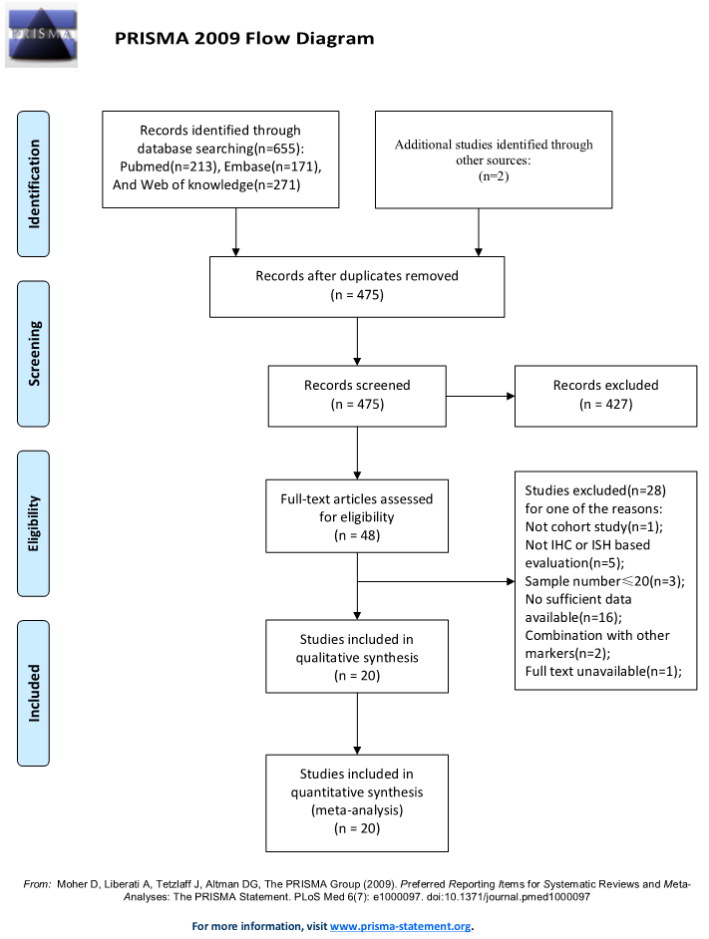
Flow diagram of the study selection process

A total of 655 potential relevant studies were retrieved after the primary database searches and two additional studies were obtained from the reference lists. After careful screening of the titles and abstracts, 609 articles were excluded as shown in Figure 1. Then eventual 48 publications underwent elaborately full-text evaluation. Eventually, 20 observational studies consisting of 2064 cases were satisfied for subsequent pooling calculation [15-19, 26-40].

### Demographic characteristics of included studies

Altogether, the sample-size ranged from 28 to 357, with a median of 103.2 participants. The median age of patients in all studies was 55.6 years old (range, 42.3-66.3) and the mean proportion of male patients was about 52.9%. The matching criteria varied among the enrolled studies. Studies concerning MEC and PTC occupied the largest proportion of cancer types among all primary literatures (*n* = 5, respectively), followed by HNSCC (*n* = 4), OSCC (*n* = 3) and remaining types of solid neoplasm. The MUC1 and MUC4 expression were evaluated by IHC staining in paraffin-embedded tissue blocks using several monoclonal antibodies as DF3, Ma695, VU4H5 for MUC1 and 1G8, 8G7, 15H10 for MUC4 respectively. As for the clinicopathological factors, seventeen eligible studies were divided into seven subgroups: 5 for age, 11 for gender, 10 for TNM tumor stage, 11 for tumor size, 13 for lymph node metastasis, 12 for tumor differentiation and 6 for mode of invasion. With respect to the quality of the including studies, none of the eligible entries scored less than six stars by the Newcastle-Ottawa Scale system, indicating a relative high methodological quality across all studies. Other detailed information were extracted and summarized in Table 1.

**Table 1:** Characteristics of eligible studies included in the meta-analysis

	First Author	Year	Country	Case Number (positive rate)	Cancer Type	Mean age	Male/ Female	AT*	Method	Ab used	Expression Location	Cutoff	C. Features^#^	Prognosis	Analysis Method	NOS** Score
1	Nitta T	2000	Japan	77 (59.7%)	OSCC	NA	NA	No	IHC	DF3 /-	Membranes and/ or cytoplasm	5%	D,T,N,S,I	NA	NA	8
2	Alos L	2005	Spain	40 (100%/ 95%)	MEC	53.0	18/22	AR/AC	IHC	Ma695 / Polyclonal	membranes and cytoplasm	5%	D	sur. curve	M	8
3	Handra LA	2005	France	63 (71%/ 79%)	MEC	52.2	20/43	NA	IHC	Ma695 /1G8	membranes and/or cytoplasm	Median	P	sur. curve	M	8
4	Wreesmann VB	2004	USA	74 (31%)	PTC	NA	28/46	No	IHC	VU4H5 /-	membranes and cytoplasm	Score≥2	NA	HR	M	7
5	Hamada T	2012	Japan	206 (39%)	OSCC	66.3	133/73	No	IHC	DF3 /-	membranes and/ cytoplasm	5%	G,D,T,N,S,P,I	HR	U/M	8
6	Cros J	2013	France	114 (26.3%)	MSGT	52+18	NA	AR/AC	IHC	Polyclonal /-	cytoplasm	Score≥2	NA	HR	U	8
7	Macha MA	2016	USA	87 (78%)	HNSCC	59.5	61/25	NA	IHC	-/ 8G7	cytoplasm	Score≥1	G,D,N,S	NA	NA	8
8	Morari EC	2010	Brazil	289 (75.8%)	PTC and FC	NA	NA	NA	IHC	VU4H5 /-	cytoplasm	Score≥3	G,T,S,A,I	NA	NA	7
9	Baek SK	2007	Korea	87 (72%)	PTC	48.6	15/72	NA	IHC	VU4H5 /1G8	membranes and/or cytoplasm	Score≥3	G,T,N,S,A,I	NA	NA	8
10	Croce MV	2008	Argentina	125 (80%)	HNSCC	59.9	98/27	NA	IHC	CT33 /-	membranes and/or cytoplasm	Median	G,D,T,N,S	NA	NA	7
11	LI X	2005	China	59 (66.1%)	HC	58.2	48/11	No	IHC	NA	membranes and cytoplasm	50%	D,N,S	NA	NA	6
12	Siyi L	2014	China	62 (92%)	MEC	56.2	48/14	No	IHC	Nov lab /-	membranes and/or cytoplasm	25%	G,T,N,P	HR	M	8
13	Renaud F	2014	France	94 (49%)	PTC	42.3	23/71	NA	IHC	M8 /-	membranes and/or cytoplasm	20%	G,N,A,I	sur. curve	M	8
14	Liu S	2014	China	357 (69%)	MEC	45.9	162/195	No	IHC	Nov lab /-	membranes and/or cytoplasm	25%	G,D,T,P	HR	M	8
15	Paleri V	2004	UK	30 (43%)	LSCC	66.0	NA	AR	ISH	NA	Nucleus and/or cytoplasm	Score≥1	NA	HR	M	6
16	Hamada T	2012	Japan	150 (41%)	OSCC	64.5	97/53	No	IHC	-/ 8G7	membranes and/or cytoplasm	5%	G,D,N,S,T,I	HR	U/M	8
17	He F	2009	China	53 (57%)	PTC	50±11/ 45±12	16/37	No	IHC	DF3 /-	membranes and/or cytoplasm	25%	G,N,A,S	NA	NA	7
18	Weed DT	2001	USA	40 (85%)	HNSCC	61	NA	No	IHC	-/ 15H10	membranes and/or cytoplasm	10%	D,T,N,S	NA	NA	7
19	Weed DT	2004	USA	28 (54% for 1G8, 79% for 15H10)	MEC	54.3	11/17	No	IHC	-/ 1G8, 15H10	membranes and/or cytoplasm	30%	D	sur. curve	M	7
20	Croce MV	2001	UK	29 (69%)	HNSCC	58.1	23/6	No	IHC	C595 /-	membranes and/or cytoplasm	Median	S,N,T,G,A	NA	NA	8

*AT: Adjuvant therapy; ^#^C. Features: clinicopathological features; AR: adjuvant radiotherapy; AC: adjuvant chemotherapy; NA: not available

**NOS: Newcastle-Ottawa Scale; Sur. curve: survival curve; IHC: immunohistochemistry; ISH: in situ hybridization

HR: hazard ratio; A: age; G:gender; T: TNM stage; D: differention; S: tumor size; N: lymph node metastasis; P: perineural invasion; I: mode of invasion

OSCC: oral squamous cell carcinoma; MEC: mucoepidermoid carcinoma; PTC: papillary carcinoma; MSGT: malignant tumors of the salivary glands

FC: follicular carcinoma; HNSCC: head and neck suqamous cell carcinoma; HC: hypopharyngeal carcinoma; LSCC: laryngeal squamous cell carcinoma

### Impact of MUC expression on survival rates of patients in HNC

**Figure 2:**
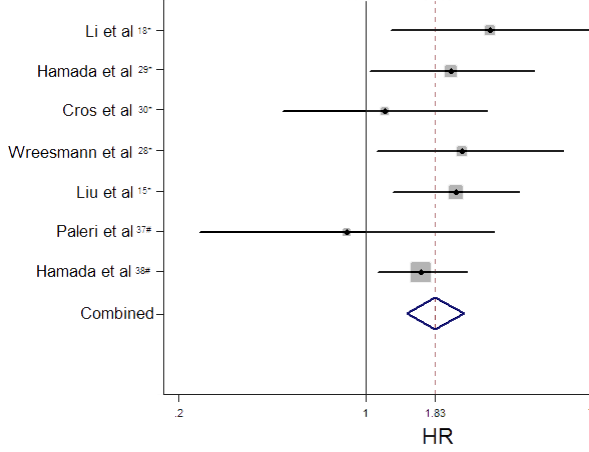
Funnel plot of hazard ratios (HR) for overall survival associated with high level of MUC expression

Seven observational trials including 993 participants offered original data on survival rates in terms of different MUC expression (Table 2). It demonstrated that increased MUC activity was associated with unfavorable survival rate (HR = 1.83, 95%CI: 1.43-2.33, *P* < 0.001). There was no significant heterogeneity among these studies (I^2^ = 0.0%, *p* (Q-test) = 0.577), so that a fixed-effect model was used to combine the HR and 95% CI (Figure 2).

**Table 2:** Results of overall and subgroup analyses for effects of MUC expression on overall survival in head and neck cancer

Categories	Study N.	Samples	Pooled data		Test of heterogeneity		
			HR (95% CI)	*p* value	Chi^2^	*p* value	I^2^ (%)
OS	7	993	1.83 (1.43-2.33)	0.000	4.74	0.577	0.0
Nationality							
Asia	4	775	1.92 (1.46-2.52)	0.000	1.95	0.583	0
Western countries	3	218	1.50 (0.88-2.57)	0.140	2.14	0.343	6.5
Cancer type							
Salivary tumors	3	533	2.04 (1.36-3.07)	0.001	2.22	0.329	10
Non-salivary tumors	4	460	1.72 (1.27-2.33)	0.000	2.07	0.557	0
Adjuvant therapy							
AR and/or AC	2	144	1.06 (0.51-2.19)	0.874	0.17	0.677	0
Non-AT	5	849	1.96 (1.51-2.53)	0	2.12	0.714	0
Sample size							
> 100	4	827	1.76 (1.34-2.31)	0	1.78	0.62	0
< 100	3	166	2.12 (1.25-3.61)	0.006	2.59	0.275	22.6
Methods							
IHC	6	963	1.88 (1.47-2.41)	0	3.29	0.656	0
ISH	1	30	0.85 (0.24-3.02)	0.802	0.00	—	—
Cut-off value							
>10%	5	607	1.95 (1.38-2.77)	0	4.08	0.395	2
<10%	2	386	1.72 (1.22-2.41)	0.002	0.39	0.535	0
MUC subtype							
MUC1	5	813	2.09 (1.51-2.89)	0	2.29	0.683	0
MUC4	2	180	1.53 (1.06-2.22)	0.023	0.91	0.341	0
Tumor location							
OSCC	2	356	1.72 (1.22-2.41)	0.002	0.39	0.535	0
LSCC	1	30	0.85 (0.24-3.02)	0.802	0.00	—	—
Antibody for MUC1							
DF3	3	625	2.28 (1.55-3.36)	0	0.42	0.812	0
Others	2	188	1.70 (0.94-3.06)	0.081	1.2	0.273	16.7
Antibody for MUC4							
8G7	1	150	1.62 (1.12-2.41)	0.014	0.00	—	—
Others	1	30	0.85 (0.24-3.02)	0.802	0.00	—	—

Furthermore, subgroup analysis stratified by nationality, cancer type, adjuvant therapy, sample size, methods, cut-off value, MUC subtype, tumor location, antibody for MUC1 and antibody for MUC4 were performed respectively.

With regard to nationality, a worse survival rate was strongly linked to MUC positivity in Asian patients (*n* = 4, HR = 1.92, 95% CI: 1.46-2.52, *p* = 0.000, I^2^ = 0.0%), while over-expression of MUC in western countries was irrelevant with poor prognosis (*n* = 3, HR = 1.50, 95% CI: 0.88-2.57, *p* = 0.140, I^2^ = 6.5%).

In the subgroup analysis by cancer type, a worse survival rate was strongly linked to MUC positivity in salivary tumors (*n* = 3, HR = 2.04, 95% CI: 1.36-3.07, *p* = 0.001, I^2^ = 10.0%) than in non-salivary tumors (*n* = 4, HR = 1.72, 95% CI: 1.27-2.33, *p* = 0.000, I^2^ = 0.0%).

As to adjuvant therapy (AT) and surgery operation, elevated MUC expression was referred to a worse prognostic role in non-AT (*n* = 5, HR = 1.96, 95% CI: 1.51-2.53, *p* = 0.000, I^2^ = 0.0%). Nevertheless, higher MUC activity was irrelevant with AT (*n* = 2, HR = 1.06, 95% CI: 0.51-2.19, *p* = 0.874, I^2^ = 0.0%).

In the subgroup analysis by sample size, higher MUC expression status was significantly associated with poorer survival rate both in sample size > 100 group (*n* = 4, HR = 1.76, 95% CI: 1.34-2.31, *p* = 0.000, I^2^ = 0.0%) and sample size < 100 group (*n* = 3, HR = 2.12, 95% CI: 1.25-3.61, *p* = 0.006, I^2^ = 22.6%).

There were two stratified subgroup in terms of detecting method among papers. Higher MUC expression was related to poorer survival rate according to IHC method (*n* = 6, HR = 1.88, 95% CI: 1.47-2.41, *p* = 0.000, I^2^ = 0.0%), but not to ISH method (*n* = 1, HR = 0.85, 95% CI: 0.24-3.02, *p* = 0.802).

Subgroups analysis by different cut-off values indicating that high MUC expression was associated to a worse prognosis no matter in cut-off > 10% (*n* = 5, HR = 1.95, 95% CI: 1.38-2.77, *p* = 0.000, I^2^ = 2.0%) or cut-off < 10% (*n* = 2, HR = 1.72, 95% CI: 1.22-2.41, *p* = 0.002, I^2^ = 0.0%).

Stratified by MUC subtype of the included papers, MUC1 positive status was identified as a worse prognosis marker in HNC (*n* = 5, HR = 2.09, 95% CI: 1.51-2.89, *p* = 0.000, I^2^ = 0.0%) than MUC4 (*n* = 2, HR = 1.53, 95% CI: 1.06-2.22, *p* = 0.023, I^2^ = 0.0%).

With regard to the tumor location, higher MUC expression was related to poorer survival rate in oral squamous cell carcinoma (OSCC) (*n* = 2, HR = 1.72, 95% CI: 1.22-2.41, *p* = 0.002, I2 = 0.0%), but not in laryngeal squamous cell carcinoma (LSCC) (*n* = 1, HR = 0.85, 95% CI: 0.24-3.02, *p* = 0.802).

As for the type of antibody used for detecting MUC1 specificity, a worse survival rate was strongly linked to DF3 antibody (*n* = 3, HR = 2.28, 95% CI: 1.55-3.36, *p* = 0.000, I^2^ = 0.0%), while over-expressions of MUC1 detected by other antibodies were irrelevant with poor prognosis (*n* = 2, HR = 1.70, 95% CI: 0.94-3.06, *p* = 0.081, I^2^ = 16.7%).

Similarly, elevated MUC4 expression was referred to a worse prognostic role detected by 8G7 antibody (*n* = 1, HR = 1.62, 95% CI: 1.12-2.41, *p* = 0.014), but not by other antibody (*n* = 1, HR = 0.85, 95% CI: 0.24-3.02, *p* = 0.802).

### Correlation of MUC expression with clinicopathological parameters

**Figure 3:**
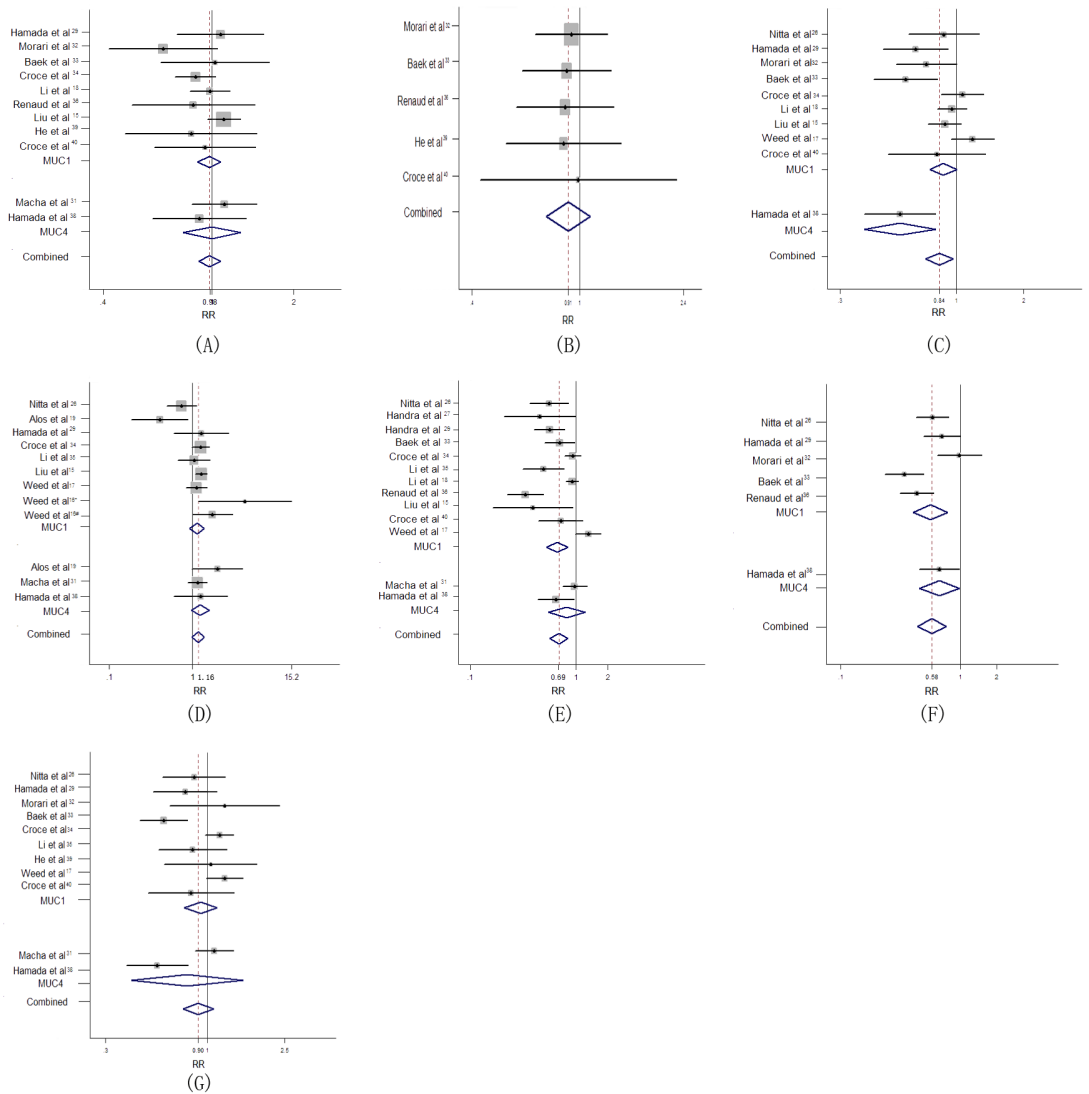
Forest plot of association between MUC overexpression with poor clinicopathological outcome in HNC (**A**) Gender (**B**) Age (**C**) UICC stage (**D**) Differentiation (**E**) Lymph node metastasis (**F**) Mode of invasion (**G**) Tumor size.

We also investigated the association of high MUC expression and clinicopathological features. As reported in Table 3, higher MUC expression was significantly associated with more advanced TNM stage (I+II *vs*.III+IV, *n* = 10, RR = 0.84, 95% CI: 0.73-0.97, *p* = 0.017, Figure 3C), lymph node metastasis (negative *vs*. positive, *n* = 13, RR = 0.69, 95% CI: 0.57-0.84, *p* = 0.000, Figure 3E), and mode of invasion (1 to 3 *vs*. 4c + 4d, *n* = 6, RR = 0.58, 95% CI: 0.44-0.76, *p* = 0.000, Figure 3F). However, MUC over-expression was not significantly associated with gender (male *vs*. female, *n* = 11, RR = 0.98, 95% CI: 0.90-1.08, *p* = 0.707), age ( < 45 yr *vs*. > 45 yr, *n* = 5, RR = 0.91, 95% CI: 0.75-1.09, *p* = 0.304), grade of differentiation (well + moderate *vs*. poor, *n* = 12, RR = 1.16, 95% CI: 0.98-1.37, *p* = 0.075) and tumor size (T1 + T2 *vs*. T3 + T4, *n* = 11, RR = 0.90, 95% CI: 0.75-1.07, *p* = 0.236).

**Table 3:** Meta-analysis of the association between MUC expression and clinicopathological features of head and neck cancer

Clinicopathological variables	Study N.	Samples	Stat.	Pooled data		Test of heterogeneity		
				RR (95% CI)	*p* value	Chi^2^	*p* value	I^2^ (%)
Gender (male/female)								
All studies	11	1502 (726/776)	FEM	0.98 (0.90-1.08 )	0.707	9,52	0.392	6.2
Subgroup								
MUC1	8	1266 (568/698)	FEM	0.98 (0.89-1.08)	0.676	8.53	0.272	0.0
MUC4	2	236 (158/78)	FEM	1.00 (0.79-1.28)	0.993	0.86	0.443	0.0
Age (<45 yr / >45 yr)								
All studies	5	514 (239/275)	FEM	0.91 (0.75-1.09)	0.304	0.12	0.998	0.0
Subgroup								
MUC1	5	514 (239/275)	FEM	0.91 (0.75-1.09)	0.304	0.12	0.998	0.0
MUC4	0	—	—	—	—	—	—	—
UICC stage (I+II/ III+IV)								
All studies	10	1296(806/490)	REM	0.84 (0.73-0.97)	0.017	26.76	0.002	66.4
Subgroup								
MUC1	9	1146(702/444)	REM	0.87 (0.76-1.00)	0.052	20.2	0.010	60.4
MUC4	1	150(104/46)	REM	0.56 (0.39-0.80)	0.002	0.00	—	—
Differentiation (well + moderate / poor)								
All studies	12	1233(902/331)	REM	1.16 (0.98-1.37)	0.075	21.89	0.025	49.7
Subgroup								
MUC1	9	961(690/271)	REM	1.12 (0.90-1.38)	0.272	19.74	0.011	59.5
MUC4	3	272(212/60)	REM	1.23 (0.96-1.58)	0.099	2.06	0.357	2.90
Lymph node (with / without metastasis)								
All studies	13	1101(424/677)	REM	0.69 (0.57-0.84)	0	58.22	0.000	79.4
Subgroup								
MUC1	11	871(338/533)	REM	0.66 (0.53-0.83)	0	55.07	0.000	81.8
MUC4	2	230(86/144)	REM	0.82 (0.55-1.22)	0.317	2.89	0.089	65.4
Mode of invasion (1 to 3 / 4c + 4d)								
All studies	6	745(498/247)	REM	0.58 (0.44-0.76)	0	18.25	0.003	72.6
Subgroup								
MUC1	5	595(389/206)	REM	0.56 (0.40-0.78)	0.001	17.42	0.002	77.0
MUC4	1	150(109/41)	REM	0.67 (0.46-0.97)	0.036	0.00	—	—
Tumor size (T1 + T2 / T3 + T4)								
All studies	11	1046(660/386)	REM	0.90 (0.75-1.07)	0.236	33.37	0.000	70.0
Subgroup								
MUC1	9	816(501/315)	REM	0.92 (0.76-1.12)	0.416	23.21	0.003	65.5
MUC4	2	230(159/71)	REM	0.79 (0.41-1.52)	0.477	9.44	0.002	89.4

Subgroup analysis stratified by different MUC subtype was performed to compare the sensitivity of MUC subtype as biomarkers. With regard to UICC stage, elevated MUC4 expression was referred to play a worse prognostic role in head and neck cancers (*n* = 1, RR = 0.56, 95% CI: 0.39-0.80, *p* = 0.002) than MUC1 (*n* = 9, RR = 0.87, 95% CI: 0.76-1.00, *p* = 0.052). For lymph node metastasis, higher MUC1 expression was related to poor prognosis (*n* = 11, RR = 1.40, 95% CI: 1.14-1.72, *p* = 0.002), but MUC4 was not (*n* = 2, RR = 1.23, 95% CI: 0.82-1.83, *p* = 0.317). Regarding mode of invasion, a worse prognostic role was strongly linked to MUC1 positivity in HNC (*n* = 5, RR = 0.56, 95% CI: 0.40-0.78, *p* = 0.001) and analogical trend was observed in aberrant MUC4 expression (*n* = 1, RR = 0.67, 95% CI: 0.46-0.97, *p* = 0.036) (Table 3).

### Sensitivity analysis

**Figure 4:**
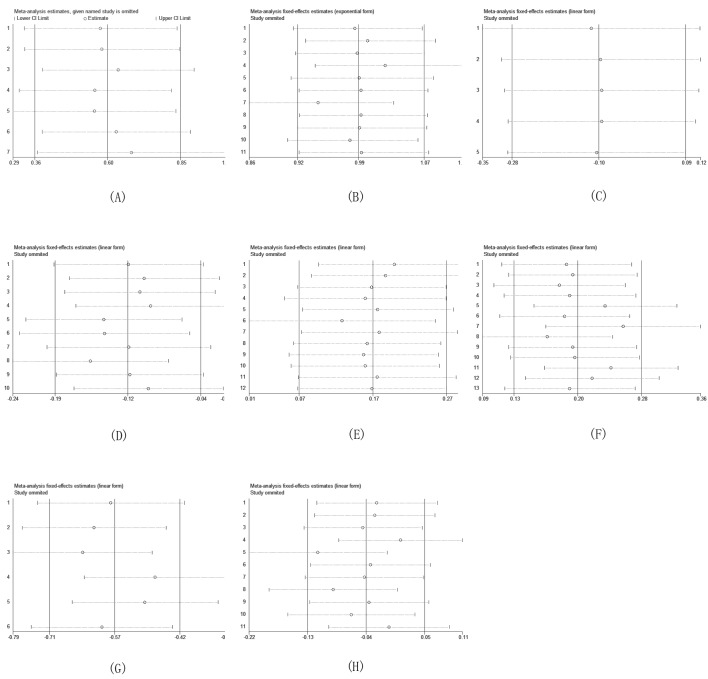
Sensitivity analysis based on stepwise omitting one study at a time for overall survival (OS) (**A**) HR (**B**) Gender (**C**) Age (**D**) UICC stage (**E**) Differentiation (**F**) Lymph node metastasis (**G**) Mode of invasion (**H**) Tumor size.

Pooled estimates of the relation of MUC overexpression to prognostic outcomes and clinicopathological parameters were not substantially altered according to the ‘leave-one-out’ method, demonstrating the reliability of our results (Figure 4A-4H).

### Publication bias

**Figure 5:**
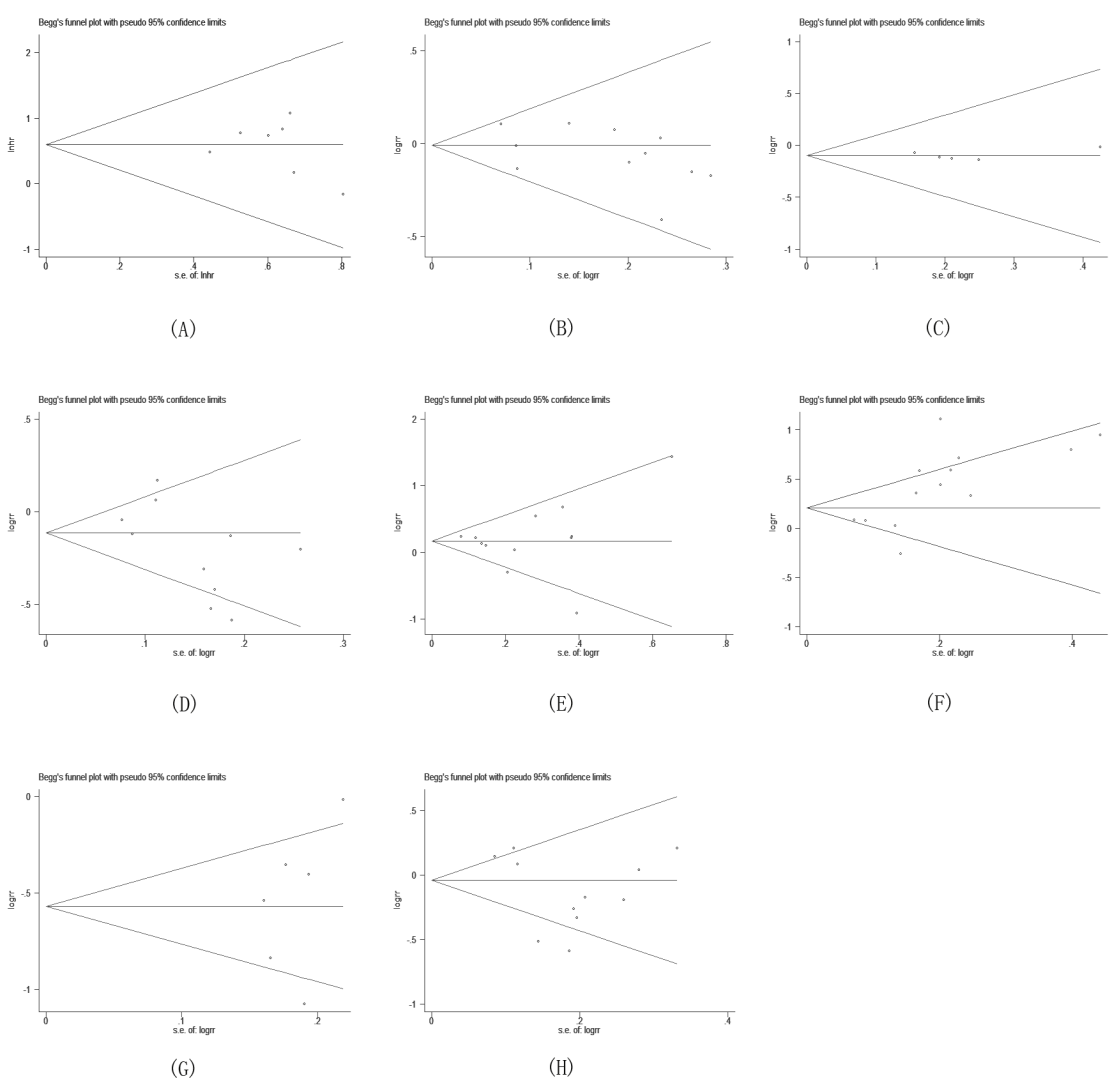
Begg's funnel plot for the evaluation of potential publication bias on overall estimate of overall survival (OS) (**A**) HR (**B**) Gender (**C**) Age (**D**) UICC stage (**E**) Differentiation (**F**) Lymph node metastasis (**G**) Mode of invasion (**H**) Tumor size.

Analysis of survival rate and clinicopathological features demonstrated no obvious asymmetry in the funnel plots for publication bias (*p* > 0.05) (Figure 5A-5H). More sensitive Egger's regression test confirmed these results, indicating that our pooled results had no significant publication bias.

### Trial sequential analysis (TSA)

**Figure 6:**
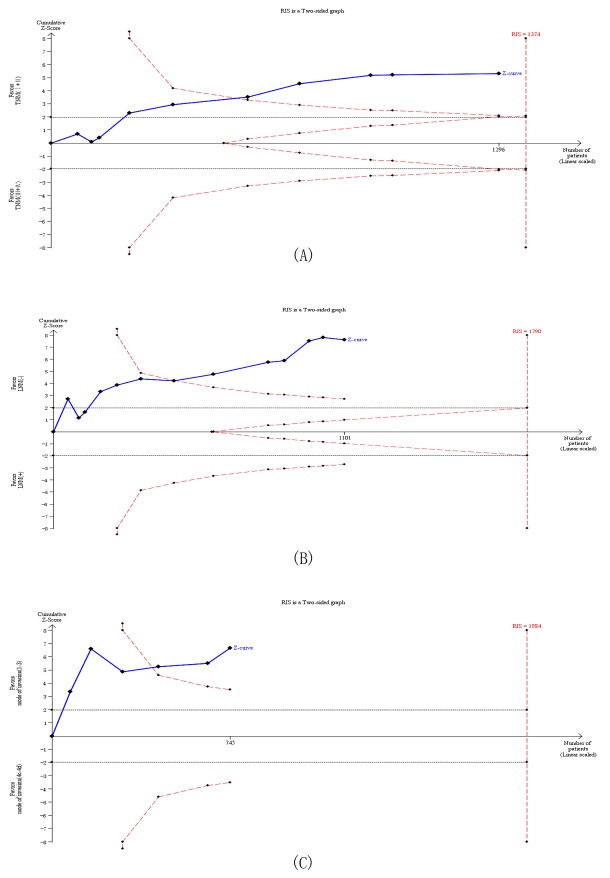
Trial sequential analysis of studies reporting the association between MUC protein expression and (**A**) UICC stage, (**B**) Lymph node metastasis and (**C**) Mode of invasion. The solid blue line represents the cumulative Z-curve. The dashed red line represents the trial sequential monitoring boundary. TSA indicates that no further trials are required..

Data from twenty trials (2046 cases) were used to investigate the reliability of MUC status as a candidate for predicting the prognosis of HNC (Figure 6A-6C). Using the relevance between MUC expression and cervical lymph node metastasis of HNC (including 13 trials with 1101 patients) as an example, the required information size (RIS) for adequate power was 1790 subjects. The cumulative z-curve crossed both the conventional boundary and the trial sequential monitoring boundary before reaching the RIS, indicating that our findings were conclusive and further trials seem to be unnecessary (Figure 6B). Similar method was applied for other groups which were not shown. On the whole, potent evidence suggested that the overexpression of MUC predicted a worse outcome.

## DISCUSSION

As revealed by the laboratorial evidence, aberrant overexpression, mislocation profiles and truncated glycans of tumor-associated MUC (TA-MUC) were commonly observed in variety of epithelial cancers. The structural and functional complexity of TA-MUC emphasizes its pivotal value in the pathogenesis and progression of cancer. However, some conflicting conclusions were reported in clinic. Although the majority of studies demonstrated that high level of MUC indicated worse clinicopathological parameters and poor prognosis, there are still a number of studies which showed the opposite conclusion [16, 17, 19]. Thus, a comprehensive study is urgently demanded.

To our knowledge, the present study is the first and most full-scale meta-analysis systemically exploring the possible prognostic role of MUC up-regulation in HNC. Here, 20 eligible studies including 2064 cases were combined to yield statistics, indicating that higher MUC expression was strongly related to poorer overall survival (HR = 1.83, 95% CI: 1.43-2.33, *p* = 0.000) in patients with HNC. Subgroup analysis was consistent with the pooled OS regardless of cancer type, sample size, staining cut-off value and MUC subtypes. Among patients accepted adjuvant therapy (AR and/or AC) along with surgery operation, no significant association was identified between positive MUC expression and poor OS, exhibiting a sharp contrast with the patients without AT (HR = 1.96, 95% CI: 1.51-2.53, *p* = 0.000). The discrepancy may be, partially, explained by the advanced radiotherapy technique and platinum-based therapeutic regimen for advanced HNC, achieving a significant improvement in 5-year OS [41]. As for western countries and ISH subgroups stratified by nationality and detecting methods respectively, no significant association was identified though a tendency was shown. This situation was possibly due to the relatively limited studies enrolled in the subgroups. As shown in Table 2, only three qualified studies for western countries and one for ISH detecting method studies were subjected to subgroup analysis, which may affect the real results. Based on these points, large-scaled studies are still needed to strength our results in the future. Stratified by the location of tumor, aberrant expression of MUC was significantly associated with poor OS in OSCC subgroup, which exhibited a sharp contrast with the patients that suffering from LSCC. The discrepancy may be, partially, explained by the morphologic diversity between oral cavity and laryngeal regions combined with different carcinogens. The inherent biological heterogeneity induces different disease evolution process though both of them originate from epithelial cells. Varying results were noted with regard to different antibodies used for detecting MUC1 or MUC4 specificity. DF3 for MUC1 may be a more tumor-specific antibody in immunohistochemistry than others (i.e. VU4H5) in HNC especially for paraffin-embedded tissues, as the same to 8G7 for MUC4. Perhaps the best explanation for staining sensitivity differences among these antibodies may be the different binding epitopes. For example, aberrantly underglycosylated TA-MUC1 tissues may somehow mask the carbohydrate epitopes recognized by VU4H5, while still permitting reaction with DF3 which binds to peptide epitopes within the variable number of tandem repeat (VNTR) domain [42]. Although different survival rate were exhibited by these antibodies, the same tendency revealed that the increased expression of MUC protein was associated with unfavorable OS in patients with HNC.

Consistent with the theoretical inference, our results also demonstrated that overexpression of MUC was tightly associated with advanced TNM stage (I+II *vs*.III+IV, RR = 0.84, 95%CI: 0.73-0.97, *p* = 0.017 ), high risk of cervical lymph node metastasis (negative *vs*. positive, RR = 0.69, 95%CI: 0.57-0.84, *p* = 0.000 ) and the depth of invasion (1 to 3 *vs*. 4c+4d, RR = 0.58, 95%CI: 0.44-0.76, *p* = 0.000 ). Given that more advanced TNM stage, more positive cervical lymph node metastasis and deeper invasion are adverse prognostic features, the pooled analysis may explain why positive MUC expression was associated with poor survival rate in patients with HNC. Several studies have uncovered unique roles of the MUC on the oncogenic and pro-metastasis effects, including promoting proliferation, metabolism, angiogenesis, invasion, metastasis, epithelial to mesenchymal transition (EMT) and resistance to apoptosis [9, 14, 43-47]. For example, multiple studies provide direct evidence for the role of MUC1 in EMT process, which enable cancer cells acquire their invasive and metastasis potential. Notably, MUC1 can directly inhibit the expression of E-cadherin, but upregulation the EMT inducers, such as Snail, Slug, Vimentin and Twist [43-44]. Similarly, MUC4 can specifically potentiate phosphorylation of the receptor tyrosine kinase ErbB2, which results in up-regulation of the cyclin-dependent kinase inhibitor p27^kip^ and silence of protein kinaseB/Akt pathways [47]. Taken together, these mechanisms can explain the association between high MUC expression and adverse clinicalpathological features in HNC patients. Therefore, MUC can serve as a good candidate for predicting the status and prognosis of HNC, making up the deficiency of the current physical detecting methods.

Apart from the inspiring outcomes, several limitations still existed in this meta-analysis. First, the lack of unified standardized protocol and evaluation system for detecting MUC status influence the accurate estimation of prognosis for HNC. Second, the numbers of studies and patients pooled into analysis were limited due to insufficient data. Third, the missing information from negative studies which were less frequently published could lead to publication bias, although significant heterogeneity was not detected from the current analysis. Finally, despite the usage of random-effect model and subgroup analysis, some heterogeneity still existed, which may weaken our pooled conclusions.

In conclusion, despite the abovementioned limitations, our meta-analysis indicated that MUC expression was significantly associated with poorer OS, more advanced TNM stage, higher risk of cervical lymph node metastasis, and deeper invasion in patients with HNC for the first time. These findings revealed that the status of MUC could not only distinguish normal and precancerous cells but also could differentiate of a less metastatic cancer form its highly aggressive form. Therefore, patients with highly expression of MUC in HNC may need more radical treatment and rigorous monitoring, especially for those without typical symptoms or signs of metastasis. However, prospective clinical trials that are well-designed, using standardized methods, with long-term follow up, are required to verify the MUC hypothesis in HNC directly.

## MATERIALS AND METHODS

### Search strategy

This meta-analysis was conducted according to the PRISMA statement [20]. A systematic literature search of PubMed, Embase, and Web of Science database was performed with the following strategy: (“mucin-1” OR “muc-1” OR “episialin”) AND (“oral” OR “mouth” OR “tongue” OR “gingival” OR “pharynx” OR “larynx”) AND (“tumor” OR “cancer” OR “carcinoma” OR “neoplasm” OR “malignant”) AND (“prognostic” OR “prognosis” OR “outcome” OR “survival”) up to September 1, 2016. Citation lists of retrieved articles, including review articles, were additionally reviewed to guarantee the sensitivity of the search process.

### Inclusion/exclusion criteria

The criteria for inclusion were listed as following: (1) studies detected MUC expression by immunohistochemistry (IHC) or *in situ* hybridization (ISH) analysis in human cancer tissues; (2) sufficient information was provided to evaluate the relationship between MUC expression and clinicopathological parameters and/or cancer prognosis; (3) the hazard ratios (HRs) with 95% confidence intervals (CIs) of overall survival (OS) were provided or can be extracted from the Kaplan-Meier curves based on the method reported before [21].

Studies were excluded if they were: (1) reviews, case reports, meta-analyses, letters, conference abstracts without original data; (2) articles which could not extract the relevant data; (3) overlapping articles and those with duplicated data.

### Data extraction

The valuable data including surname of the first author, mean age, gender, cancer type, anti-body used, clinicopathological features, prognosis and other relevant data were extracted by two investigators (Lu and Liang) independently and illustrated in Table 1. When the prognosis was only plotted as a Kaplan-Meier curve in some articles, Engauge Digital 9.3 software (from https://sourceforge.net/projects/digitizer/) was applied to digitize and extract the data. A joint decision was offered in the case of any disagreement.

### Quality assessment

The quality of included studies was assessed by two independent reviewers (Zhu and Xu) on the basis of the Newcastle-Ottawa scale (NOS) system, which is a 9-point scoring system by judging three main categories: including selection, comparability and outcome. Studies with scores≥6 were regarded as high-quality studies [22-23].

### Statistical analysis

Pooled HR with their 95% CI was used to assess the association between MUC expression levels and the cancer prognosis (OS). Meanwhile, the impact of MUC expression on clinicopathological parameters was performed by RR with their 95% CIs. The heterogeneity among the included studies was checked by the chi-squared Q test. *P* > 0.10 or I^2^ < 50% indicated that the differences between the results of various studies were due to chance, and then a fixed-effect model was used. Otherwise, a random-effects model was employed. Subgroup analyses were performed to explore the source of heterogeneity. The sensitivity analysis and funnel plots were carried out to evaluate the robustness and the possible publication bias respectively. All statistical tests were performed using Stata version 12.0 (Stata Corp, College Station, TX, USA) with two-tails *p* values. A *p*-value < 0.05 was considered to be statistically significant.

### Trial sequential analysis (TSA)

TSA was employed to test whether the current findings were reliable and conclusive, based on diversity-adjusted threshold for statistical significance. We conducted TSA with assumptions including a plausible overall 5% risk of type I error with a power of 80%. When the cumulative z-curve crosses the trial monitoring boundary or enters the futility zone before the required power is reached, future trials are superfluous considering a sufficient level of evidence is reached [24-25]. Otherwise, the current evidence is insufficient for drawing a conclusion. These analyses were performed using Trial Sequential Analysis Software version 0.9 beta (www.ctu.dk/tsa).
